# Nephrotoxic Effects of Paraoxon in Three Rat Models of Acute Intoxication

**DOI:** 10.3390/ijms222413625

**Published:** 2021-12-20

**Authors:** Vladislav E. Sobolev, Margarita O. Sokolova, Richard O. Jenkins, Nikolay V. Goncharov

**Affiliations:** 1 Sechenov Institute of Evolutionary Physiology and Biochemistry, Russian Academy of Sciences, Thorez 44, 194223 St. Petersburg, Russia; vesob@mail.ru (V.E.S.); Sokolova.Rita@gmail.com (M.O.S.); 2 Leicester School of Allied Health Sciences, De Montfort University, The Gateway, Leicester LE1 9BH, UK; roj@dmu.ac.uk

**Keywords:** nephrotoxicity, rats, paraoxon, biomarkers, calbindin, chondroitin sulphate, creatinine clearance, histopathology, morphometry, electron microscopy, glycosaminoglycans, nephrin

## Abstract

The delayed effects of acute intoxication by organophosphates (OPs) are poorly understood, and the various experimental animal models often do not take into account species characteristics. The principal biochemical feature of rodents is the presence of carboxylesterase in blood plasma, which is a target for OPs and can greatly distort their specific effects. The present study was designed to investigate the nephrotoxic effects of paraoxon (O,O-diethyl O-(4-nitrophenyl) phosphate, POX) using three models of acute poisoning in outbred Wistar rats. In the first model (*M1*, POX2x group), POX was administered twice at doses 110 µg/kg and 130 µg/kg subcutaneously, with an interval of 1 h. In the second model (*M2*, CBPOX group), 1 h prior to POX poisoning at a dose of 130 µg/kg subcutaneously, carboxylesterase activity was pre-inhibited by administration of specific inhibitor cresylbenzodioxaphosphorin oxide (CBDP, 3.3 mg/kg intraperitoneally). In the third model (*M3*), POX was administered subcutaneously just once at doses of LD16 (241 µg/kg), LD50 (250 µg/kg), and LD84 (259 µg/kg). Animal observation and sampling were performed 1, 3, and 7 days after the exposure. Endogenous creatinine clearance (ECC) decreased in 24 h in the POX2x group (*p* = 0.011). Glucosuria was observed in rats 24 h after exposure to POX in both M1 and M2 models. After 3 days, an increase in urinary excretion of chondroitin sulfate (CS, *p* = 0.024) and calbindin (*p* = 0.006) was observed in rats of the CBPOX group. Morphometric analysis revealed a number of differences most significant for rats in the CBPOX group. Furthermore, there was an increase in the area of the renal corpuscles (*p* = 0.0006), an increase in the diameter of the lumen of the proximal convoluted tubules (PCT, *p* = 0.0006), and narrowing of the diameter of the distal tubules (*p* = 0.001). After 7 days, the diameter of the PCT lumen was still increased in the nephrons of the CBPOX group (*p* = 0.0009). In the *M3* model, histopathological and ultrastructural changes in the kidneys were revealed after the exposure to POX at doses of LD50 and LD84. Over a period from 24 h to 3 days, a significant (*p* = 0.018) expansion of Bowman’s capsule was observed in the kidneys of rats of both the LD50 and LD84 groups. In the epithelium of the proximal tubules, stretching of the basal labyrinth, pycnotic nuclei, and desquamation of microvilli on the apical surface were revealed. In the epithelium of the distal tubules, partial swelling and destruction of mitochondria and pycnotic nuclei was observed, and nuclei were displaced towards the apical surface of cells. After 7 days of the exposure to POX, an increase in the thickness of the glomerular basement membrane (GBM) was observed in the LD50 and LD84 groups (*p* = 0.019 and 0.026, respectively). Moreover, signs of damage to tubular epithelial cells persisted with blockage of the tubule lumen by cellular detritus and local destruction of the surface of apical cells. Comparison of results from the three models demonstrates that the nephrotoxic effects of POX, evaluated at 1 and 3 days, appear regardless of prior inhibition of carboxylesterase activity.

## 1. Introduction

The kidneys of humans and animals are particularly susceptible to the toxic effects of chemicals because they are continuously supplied with large volumes of blood, ranging from 15 to 25% of cardiac output [1]. Renal secretion and reabsorption mechanisms providing high concentrations of blood-borne toxic compounds in the tubules or interstitium can lead to their accumulation in renal cells and tissues. Many xenobiotics cause direct damage to the tubules or glomeruli, followed by impaired secretory and reabsorptive functions or glomerular permeability. The renal cortex is the most susceptible to toxic damage because it receives the greatest blood supply; the renal cortical blood flow averages 4.67 ± 0.31 mL/g tissue/min in healthy humans [2] and 7.39 ± 0.07 mL/g tissue/min in rats [3].

Organophosphates (OPs), due to their widespread distribution in different areas of human life, including agriculture and industry, are the subject of close study of their effects on human and animal health. Over the past 60 years, OPs poisoning has become one of the most serious public health problems worldwide. The World Health Organization estimates that over three million cases of OPs poisoning occur each year. Of these, more than 250,000 are fatal [4]. The primary mechanism of OPs toxicity in humans and animals is the irreversible inhibition of cholinesterases, leading to the accumulation of acetylcholine in the synaptic cleft, causing overstimulation of nicotinic and muscarinic receptors in the central and peripheral nervous system [5]. This stimulation leads to a variety of clinical symptoms of OPs poisoning, including salivation, lacrimation, exophthalamus, diarrhoea, miosis, hypothermia, muscle fasciculations, bradycardia, bronchospasm, pulmonary edema, pneumonia, pancreatitis, and renal failure.

Damage to the kidneys and their impaired functions after a single contact with OPs, accompanied by the development of clinical manifestations of poisoning, was considered in the early 1990’s to be a rare manifestation of their toxicity [1]. At that time, only three publications mentioning nephrotoxic effects after OPs poisoning in humans had been published in English-language literature [6,7,8]. Since the beginning of the 21st century, the nephrotoxicity of OPs has been the subject of greater attention. In in vivo experiments with laboratory animals, nephrotoxicity has been established for many OPs compounds, including fenthion [4,9], diazinon [10], malathion [11,12], chlorpyrifos [13], dichlorfos [14], metamidophos [15], quinalfos [16], and methylparathion [17]. At the same time, an increasing number of publications has been devoted to the study of clinical cases of human poisoning with OPs compounds that were accompanied by nephrotoxic effects, such as acute kidney injury (AKI) and development of acute renal failure (ARF) [18,19,20].

Acute renal failure is one of the problems that appear in the clinical follow-up of patients and is responsible for the increased mortality in ARF poisoning [19,20]. Various mechanisms have been proposed to explain the development of ARF after OPs intoxication, but knowledge is limited due to insufficient experimental data. For example, it has been suggested that the development of AKI after OPs poisoning may be related to rhabdomyolysis and myoglobinuria due to muscle fasciuria and convulsions [4]. In an acidic environment, myoglobin precipitates as acidic haematin, leading to blockage of the ascending part of the Henle loops. Myoglobin, which has a toxic effect, concentrates in the distal nephron and can cause myoglobinuria nephrosis and acute tubular necrosis [21]. Damage to the tubules has, therefore, been suggested to be a major cause of renal dysfunction following OPs poisoning [4]. This hypothesis has been partially confirmed in in vivo experiments with rats using OPs, such as fenthion [4] and paraoxon [21].

Paraoxon (O,O-diethyl O-(4-nitrophenyl) phosphate, POX) is a metabolic product of ethyl parathion, and is one of the most toxic pesticides for restricted use to control a broad spectrum of pests on alfalfa, barley, canola, corn, cotton, sorghum, soybeans, sunflowers, and wheat. POX as a potent cholinesterase inhibitor is responsible for the cholinergic crisis typical of parathion poisoning [22]. According to some authors, the histopathological manifestation of nephrotoxicity of POX in rats after a single sublethal dose poisoning is disorganization of proximal tubule epithelial cells with an increase in their diameter [21]. The results of in vitro studies showed that POX is able to induce apoptosis, which was detected in T-lymphocytic leukemia EL4 cells of mice. This stimulation occurs through a direct effect of POX on mitochondria, disrupting their transmembrane potential and causing release of cytochrome *C* into the cytosol with subsequent activation of caspase-9 [23].

The toxic effects of OPs are not limited to inhibition of cholinesterases. They are also capable of producing reactive oxygen species (ROS) that damage renal tubules and renal parenchyma, leading to hypovolemia and AKI. Oxidative stress is a major complication in the formation of renal pathology in OPs poisoning. POX in rats causes production of ROS and oxidative stress manifested by GSH depletion in kidney tissue [24]. In another study, it was found that the level of ROS production after POX poisoning is dose-dependent, and the induction of oxidative stress occurs in the following order: brain > liver > heart > kidney > spleen [25]. Administration of POX to rats at doses that cause the development of systemic toxicity also leads to vasoconstriction of the kidneys and a reduction in both efficacious renal blood flow (RBF) and glomerular filtration rate (GFR) [26].

It should be noted that, with the exception of the above studies, more detailed studies of POX nephrotoxicity have not been published, and the mechanisms of its nephrotoxic effects remain unclear. In this regard, and in order to obtain new data with POX as a typical representative of OPs to elucidate the possible mechanisms of OPs nephrotoxicity in general, we studied acute toxicity in an in vivo model with outbred Wistar rats [27,28,29,30]. The present article presents and analyzes new data that directly and indirectly characterize the nephrotoxicity of POX in three models of acute rat poisoning. The main objective was to determine the most important manifestations of POX nephrotoxicity, necessary to understand the pathophysiological mechanisms in the early period after OPs poisoning, and to determine the prospects for further studies in this direction.

## 2. Results

### 2.1. Nephrotoxicity of POX in the M1 and M2 Models

#### 2.1.1. Plasma Biochemistry

Of the 27 biochemical parameters, changes in plasma creatinine, urea, and uric acid were the most likely indicators of renal dysfunction. This was particularly the case 3 h after poisoning creatinine level had already increased by 35–40% in the CBPOX group (*p* < 0.05). Uric acid content in plasma was changed only in the POX2x group, with an increase of 20–40% by 1 and 7 days after poisoning. Since up to 80% of uric acid is excreted by the kidneys, these data provide further evidence of renal damage in acute OPs poisoning. Almost synchronous increase calcium and inorganic phosphate level and alkaline phosphatase activity was found in the CBPOX group after 3 h of poisoning. 

**Figure 1:**
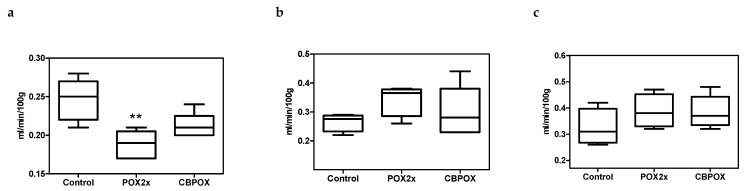
Endogenous creatinine clearance in rats: (**a**) 1 day, (**b**) 3 days, and (**c**) 7 days after the poisoning; ** *p* < 0.01 compared to Control group.

Endogenous creatinine clearance (ECC) in rats decreased markedly in 24 h of POX intoxication, regardless of the mode of cholinesterase inhibition. In the POX2x group, it was 0.18 (*p* = 0.011); in the CBPOX group, 0.21; and in the control animals, 0.25 mL/min/100 g BW. At 3 and 7 days after the poisoning, ECC was not significantly different in rats of all groups (Figure 1).

#### 2.1.2. Urinalysis

After poisoning for 1 day, the diuresis of rats was not significantly different from that of unpoisoned animals. At 3 days, there was an increase in daily urine output in the POX2x group of 9.6 ± 1.5 mL, and in the CBPOX group of 12.8 ± 1.5 mL (*p* < 0.05), compared to the control group of 8.5 ± 1.2 mL. There were no significant differences at 7 days after poisoning.

**Figure 2:**
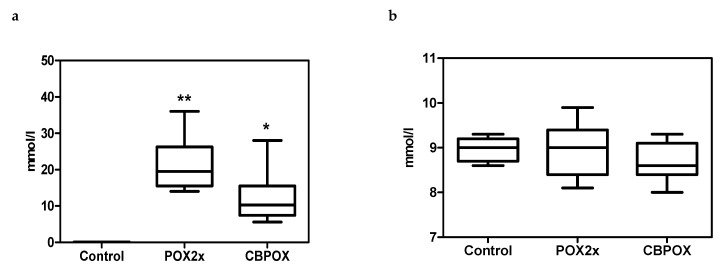
Glucose content 24 h after poisoning: (**a**) in rat urine; (**b**) in rat plasma. * *p* ≤ 0.05; ** *p* ≤ 0.01 compared to the control group.

Over the course of the experiment, urine pH in rats ranged from 6.0 to 7.0 with no statistically significant differences between groups. Specific gravity of urine also did not differ between groups and was determined in the range from 1.016 to 1.025. Microalbuminuria was detected in the urine of animals of all groups, observed over the course of the experiment at the level of 30–80 mg/L, while in the POX2x and CBPOX groups, no significant excess was found compared to the control group. The only significant difference in urine biochemistry of poisoned rats was a marked glucosuria observed 24 h after the poisoning in both POX2x and CBPOX groups (Figure 2).

Microscopic examination of rat urine sediment showed no signs of acute renal damage, including in haematuria, erythrocyturia, pyuria, crystalluria, and cylinduria, as well as increased epithelial cell count.

#### 2.1.3. Chondroitin Sulphate (CS) in Rat Urine

The content of sulphated GAG in the urine was estimated by the concentration of chondroitin sulphate (CS), normalized to creatinine in the daily urine samples. At 24 h after poisoning, the CS level in the urine tended to increase: 11.3 ± 4.4 in the POX2x group; 10.1 ± 3.6 in the CBPOX group; and 5.7 ± 0.4 mg/mol creatinine in control animals. After 3 days, the CS content in the urine of poisoned rats was still elevated: 8.6 ± 1.8 in the POX2x group; 8.3 ± 0.9 (*p* < 0.05) in the CBPOX group; and 6.1 ± 0.6 mg/mol creatinine in control animals. At 7 days after the poisoning, there was no significant difference in the urinary CS content in the poisoned rats: 5.2 ± 0.9 in the POX2x group, 6.6 ± 1.5 in the CBPOX group, and 5.9 ± 1.7 mg CS/mol creatinine in the control group.

#### 2.1.4. Calbindin, KIM-1, TIMP-1 in Rat Urine

The level of these biomarkers in the urine of poisoned rats at 1 and 7 days after the poisoning showed no significant differences from that of control animals. However, 3 days after the poisoning, a significant increase in the concentration of calbindin was observed in the CBPOX group (Table 1).

#### 2.1.5. Histopathological Changes in the Kidneys

**Figure 3:**
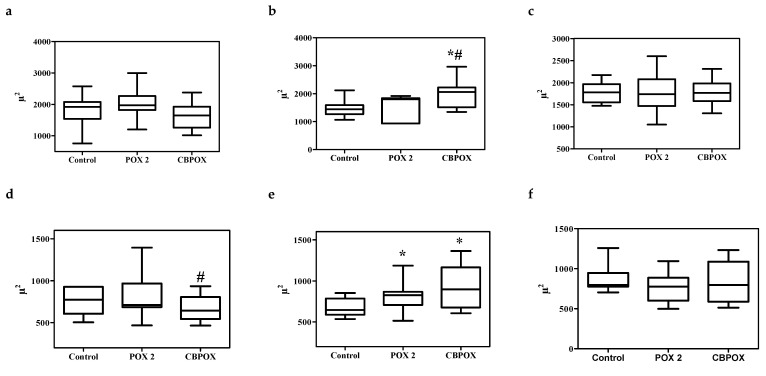
A (**a**–**c**). Renal corpuscle area, mm^2^: (**a**) 1 day, (**b**) 3 days, and (**c**) 7 days after exposure to POX. B (**d**–**f**). Renal glomerulus area, mm^2^: (**d**) 1 day, (**e**) 3 days, and (**f**) 7 days after the exposure. * *p* ≤ 0.01 compared to the control group; # *p* ≤ 0.01 between POX2x and CBPOX groups.

**Figure 4:**
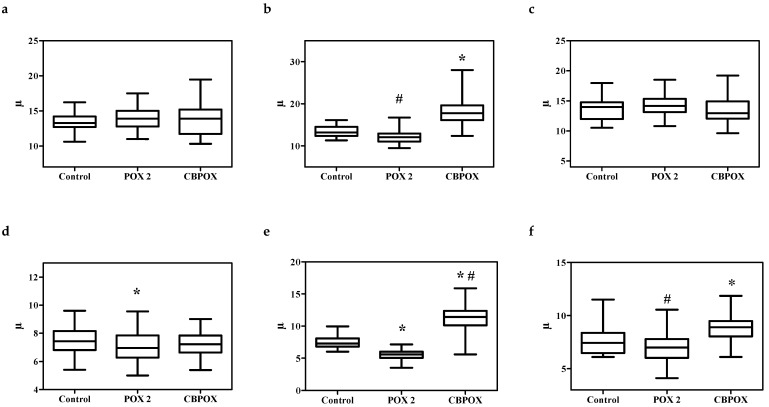
A (**a**–**c**). Diameter of proximal convoluted tubules (PCT), μm: (**a**) 1 day, (**b**) 3 days, and (**c**) 7 days exposure to POX. B (**d**–**f**). Lumen diameter of the proximal convoluted tubules (PCT), μm: (**d**) 1 day, (**e**) 3 days, and (**f**) 7 days after the exposure. * *p* ≤ 0.01 compared to the control group; # *p* ≤ 0.01 between the POX2x and CBPOX groups.

**Figure 5:**
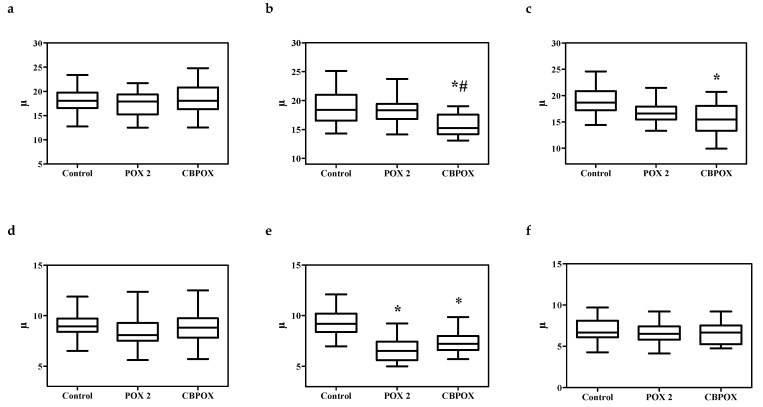
A (**a**–**c**). Diameter of distal convoluted tubules (DCT), μm: (**a**) 1 day, (**b**) 3 days, and (**c**) 7 days after exposure to POX. B (**d**–**f**). Lumen diameter of distal convoluted tubules (DCT), μm: (**d**) 1 day, (**e**) 3 days, and (**f**) 7 days after the poisoning. * *p* ≤ 0.01 compared to the control group; # *p* ≤ 0.01 between POX2x and CBPOX groups.

Examination of the histological sections of the kidneys of the poisoned animals did not reveal significant morphological differences in comparison with the control animals. In this regard, to objectively assess histological changes after exposure to POX, morphometric evaluations of renal corpuscle area, renal glomerulus area, diameters of proximal convoluted tubules, and diameters of distal convoluted tubules were performed. The results are shown in Figure 3, Figure 4 and Figure 5.

As seen from Figure 3, Figure 4 and Figure 5, within 24 h after the poisoning, there were no significant changes in the morphometry of the renal corpuscles, glomerulus, proximal, and distal convoluted tubules. After 3 days of poisoning, there was an increase in the area of the renal corpuscles and glomeruli, especially pronounced in the CBPOX group. After 3 days, lumen diameter of proximal tubules had decreased in the POX2x group and increased in the CBPOX group. The diameter of the lumen of distal tubules at this time point decreased in the poisoned rats of both groups. At 7 days after the poisoning, there were no significant differences in the morphometric indices in the animals of the POX2x group, but in the nephrons of the CBPOX group, the lumen diameter of the PCT was still enlarged.

Avoiding unnecessary speculation on the interpretation of the obtained results of morphometry of rat nephron elements, it should be noted that they are not specific enough to draw conclusions about the mechanisms of POX nephrotoxicity at the histological level. Combined interpretation of these data with the results of biochemical studies suggests that impaired renal function after a single poisoning with POX is manifested and compensated earlier than the formation of structural disorders of nephron elements. It is quite logical to assume that morphometric changes reflect the toxic effect of POX, which is fully or partially compensated by the renal homeostasis maintenance systems by day 7 after the poisoning.

Summarizing the results of studies on the *M1* and *M2* models, the greatest changes in plasma biochemical parameters, kidney damage biomarkers, and histological data were observed within 1 to 3 days after exposure to POX. In the *M1* model with prior inhibition of cholinesterase activity by CBDP, a more pronounced effect of POX for all analyzed parameters was found, and in this regard, is preferable for extrapolation of the data to humans. Table 2 summarizes the results of the signs of POX nephrotoxicity in the *M1* model.

### 2.2. Paraoxon Nephrotoxicity in the M3 Model (without Prior Inhibition of Carboxylesterase Activity)

At this stage of the study, taking into account the changes in the morphology of nephron elements revealed in the *M1* and *M2* models, the focus was on the histopathological and ultrastructural changes in rat kidneys 1, 3 and 7 days after acute exposure to POX at doses of LD16, LD50, and LD84.

#### 2.2.1. Histopathological Changes in Rat Kidneys after Exposure to POX

**Figure 6:**
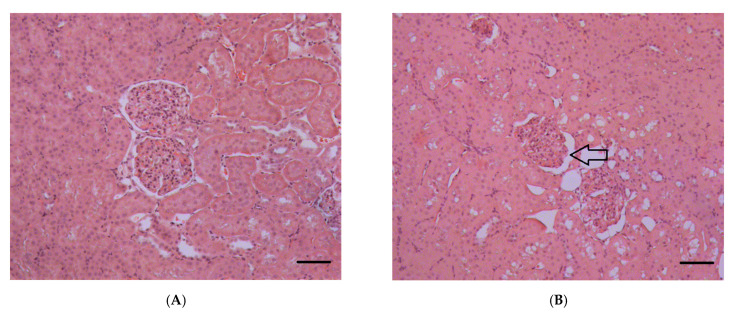

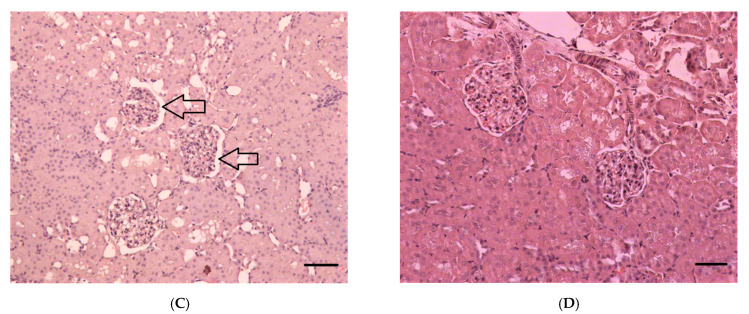
A section of the rat kidney cortex after exposure to POX at LD84. (**A**)—control, (**B**)—1 day, (**C**)—3 days, and (**D**)—7 days after the exposure. The arrows show enlargement of the urinary space. H&E stain. (×200). scale bar = 50 µm.

At the level of light microscopy imaging, poisoning at a dose of LD16 does not lead to changes in the structure of kidney tissues and cells. After POX poisoning at LD50 and LD84 levels, microscopic examination of kidney sections revealed damage to tubule epihelial cells. By 1 and 3 days after the poisoning at a dose of LD84, the cell cytoplasm had become more homogeneous, and the granularity normally present in tubule epithelial cells had disappeared. It should be noted that, after 7 days of exposure to POX, the morphological characteristics of cytoplasm were completely restored (Figure 6). One day after the poisoning, there was an increase in the diameter of the tubule lumen in the LD84 group, and after 3 days of exposure to POX, their narrowing was observed (*p* < 0.05).

**Figure 7:**
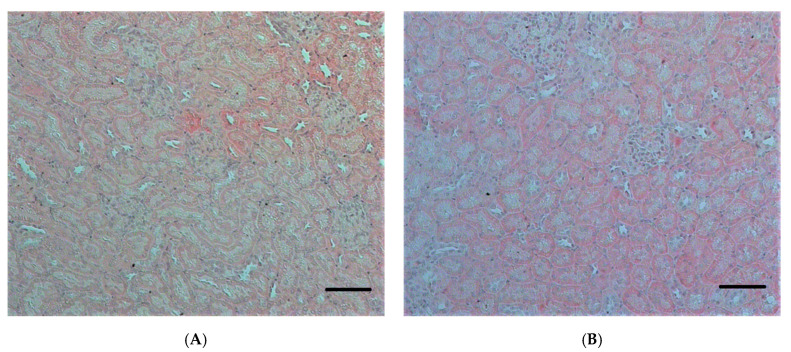

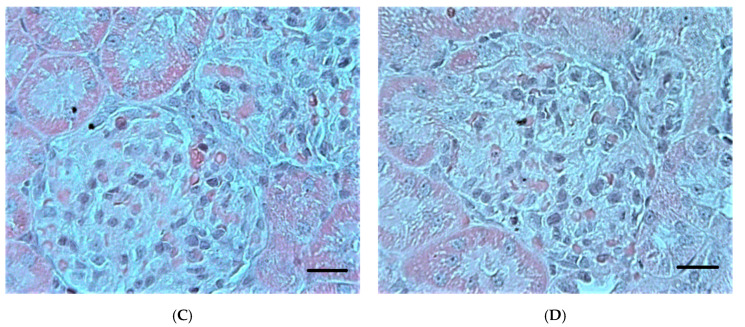
(**A**,**B**)—Expression of nephrin in the kidney cortex 24 h after exposure to POX. (**A**)—LD84; (**C**)—LD50. (**C**,**D**)—Expression of nephrin in renal cortex cells and tubules 24 h after the exposure at LD50. IHC, fast red chromogen (×200). Scale bar = 100 µm (**A**,**B**); 10 µm (**C**,**D**).

**Figure 8:**
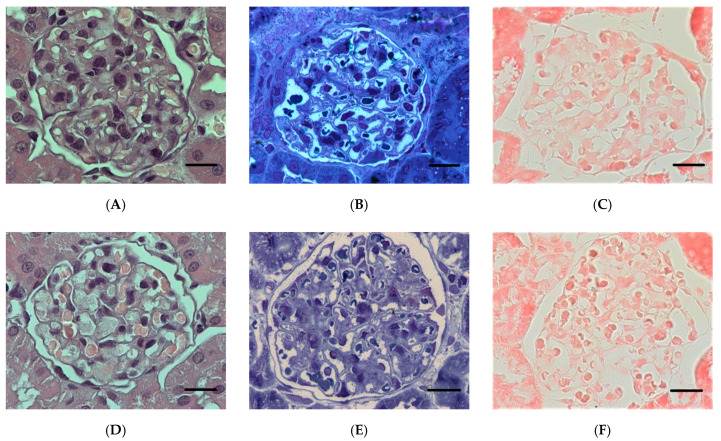
Renal cortex of rat kidney. (**A**–**C**)—control; (**D**–**F**)—24 h after exposure to POX (LD84). H&E stain (**A**,**D**); methylene blue stain (**B**,**E**); IHC to nephrin, fast red chromogen (**C**,**F**). (×1000). Scale bar = 10 µm.

**Figure 9:**
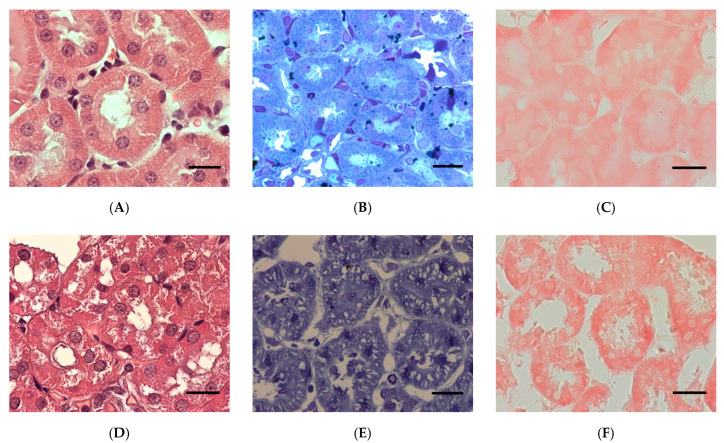
Epithelium of rat renal cortex tubules. (**A**–**C**)—control; (**D**–**F**)—24 h after exposure to POX (LD84). H&E stain (**A**,**D**); methylene blue stain (**B**,**E**); IHC to nephrin, fast red chromogen (**C**,**F**). (×1000). Scale bar = 10 µm.

The structure of the renal corpuscles 24 h after poisoning in the LD50 and LD84 groups showed a decrease in the ratio of the size of the renal corpuscles to the glomerular area (*p* < 0.05), indicating expansion of the Bowman′s capsule. Similar changes in the renal corpuscle are shown 3 days after exposure to POX, but were not observed at the 7 days’ checkpoint (Figure 6). The IHC study of nephrin showed a comparable level of expression of this protein in GBM and tubules in control and POX-poisoned rats (Figure 7, Figure 8 and Figure 9).

The revealed positive reaction to nephrin is most intensively manifested in tubule epithelial cells. The basal part of tubule epithelial cells clearly shows granularity (Figure 9). In the renal corpuscle, the cytoplasm of podocytes is weakly stained, and clear localization of nephrin at the edge of GBM is absent in all cases (Figure 8).

#### 2.2.2. Ultrastructural Changes in Rat Kidneys after the Poisoning

**Figure 10:**
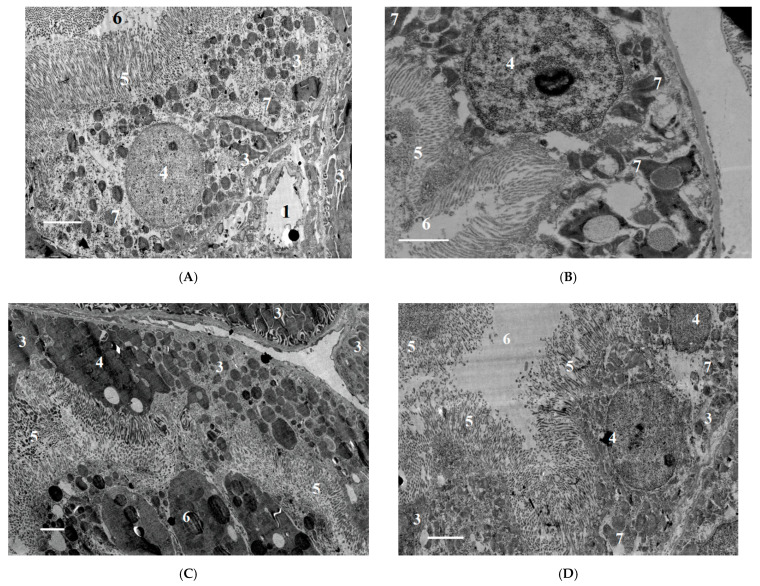
(**A**)—control; (**B**)—LD50, 24 h after exposure to POX; (**C**)—LD84, 24 h after exposure to POX; (**D**)—LD84, 3 days after exposure to POX. (**B**–**D**)—Vacuolization of cytoplasm, degeneration of mitochondria in epithelial cells of proximal tubules. (**C**)—Karyopiknosis of nuclei after acute poisoning with a dose at LD84. Increased height of proximal tubule epithelium 3 days after exposure to POX (**D**). 1—capillary lumen, 2—epithelial cell of the distal tubule, 3—epithelial cell of the distal tubule, 4—nucleus, 5—microvilli, 6—tubule lumen, 7—mitochondria. Double contrasting with lead nitrate and uranyl acetate. TEM, scale bar = 2 µm.

**Figure 11:**
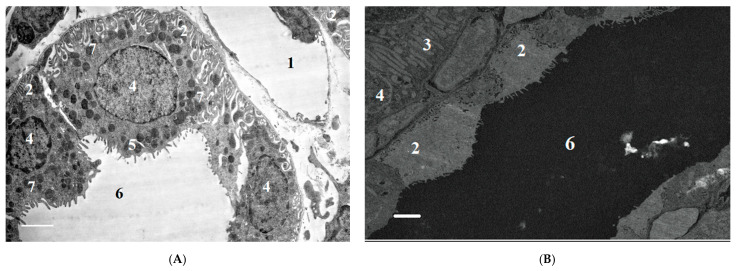

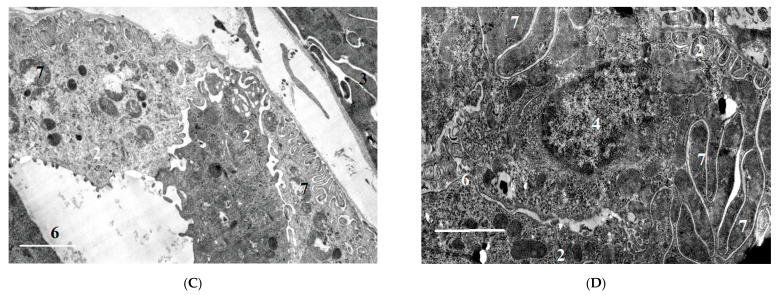
(**A**)—control; (**B**)—LD50, 24 h after exposure to POX; (**C**)—LD84, 24 h after exposure to POX; (**D**)—LD84, 3 days after exposure to POX. (**B**,**C**)—decrease in height of epithelial cells of distal convoluted renal tubules 24 h after exposure to POX (*p* < 0.05), and reduction in tubule lumen 3 days after exposure to POX (**D**) (*p* < 0.05); (**C**)—mitochondrial degradation. 1—capillary lumen, 2—epithelial cell of distal tubule, 3—epithelial cell of distal tubule, 4—nucleus, 5—microvessels, 6—tubule lumen, 7—mitochondria. Double contrasting with lead nitrate and uranyl acetate. TEM, scale bar = 2 µm.

In the LD84 group, we detected a decrease in the height of epithelial cells relative to the distal tubules 24 h after the poisoning (*p* < 0.05), and an increase in the proximal tubule epithelium 3 days after the poisoning (*p* < 0.05). In the epithelium of proximal tubules, the damages were expressed locally and were manifested as stretching of basal labyrinth, sloughing of microvilli on apical surface and presence of pycnotic nuclei in cells. Partial swelling and destruction of mitochondria, the presence of pyknotic nuclei, and the displacement of nuclei to apical cell surfaces were observed in the epithelium of distal tubules (Figure 10 and Figure 11).

After 7 days of exposure to POX at doses of LD50 and LD84, some signs of tubule epithelial cell destruction remained, in particular, the blocking of tubule lumen by cellular detritus and local destruction of apical cell surfaces. Thus, at this period after the poisoning, incomplete recovery of proximal tubule epithelium is observed.

The structure of GFB shows no signs of significant damage after the poisoning with the doses studied. The urinary space contains a small amount of cellular detritus. Electron-dense deposits along basal membranes or in mesangium were not detected, and capillary lumen was free. Morphometric analysis of GBM 1 and 3 days after the poisoning revealed no statistically significant differences. Thus, the thickness of GBM 24 h after the poisoning in animals was 0.186 ± 0.004 μm the control group; 0.193 ± 0.07 μm in the LD16 group; 0.18 ± 0.03 μm in the LD50 group; and 0.201 ± 0.04 μm in the LD84 group. However, 7 days after the poisoning, a statistically significant (*p* < 0.05) increase in GBM thickness was observed: 0.212 ± 0.06 μm in the LD50 group; 0.209 ± 0.03 μm in the LD84 group; and 0.186 ± 0.04 μm in the control group.

By summarizing the results of the *M3* model of exposure to POX, it should be noted that the LD16 dose had a minimal effect on the changes in the assessed parameters at the histological and ultrastructural level. In this regard, there is no reason to talk of a nephrotoxic effect of POX at this dose. However, as shown by the results of histopathological and EM studies, even a single ingestion of POX at doses of LD50 and LD84 leads to the development of morphological changes, especially those pronounced 1–3 days after the poisoning. As in the *M1* and *M2* models, at 7 days post-poisoning, most of these changes were compensated by the renal systems. Table 3 summarizes the results of the signs of POX nephrotoxicity in the *M3* model.

## 3. Discussion

### 3.1. Putative Pathophysiological Mechanisms of OPs Nephrotoxicity

The pathophysiological mechanisms of OPs renal damage are not fully understood and there are many assumptions in the rather limited literature on this subject [31]. The kidneys are innervated by both adrenergic and cholinergic components. Increased renal adrenergic tone may contribute to acute tubular necrosis, increased renal vascular resistance, activation of the renin–angiotensin system, or possibly increased transmembrane Ca^2+^ transport in renal tubule cells [32].

As early as the 1970s, it was shown that the kidneys contain OPs-sensitive cholinesterases [33]. Some researchers attribute OPs nephrotoxicity to high levels of pseudocholinesterase in the distal tubules of the kidneys; oxidative stress, due to high OPs concentration in the tubule lumen [34]; and rhabdomyolysis and hypovolemia, due to MODS development and dehydration [35]. It is assumed that renal blood flow and electrolyte excretion are partially controlled by cholinergic mechanism, so OPs poisoning impairs renal function [1].

Experimental data indicate that acetylcholine (ACh) directly affects renal excretory function. After ACh is injected into the renal artery, there is a significant increase in the excretion of water, K^+^, Na^+^, Cl^−^, Ca^2+^, and phosphate, and urine osmolarity decreases [36,37,38]. The proximal tubules are involved in the increase in CFR and phosphate excretion [36]. Thus, several mechanisms may be involved in the nephrotoxic effects of OPs, which will be manifested depending on the route of entry and the dose of OPs.

### 3.2. Biochemical Aspects of POX Nephrotoxicity

In studies of acute toxicity of POX in the *M 1/2* models, an increase in the content of creatinine, urea, and uric acid in the blood plasma of rats in the early period after the poisoning was registered.

Increased concentrations of creatinine and urea in plasma or serum of rats after poisoning with OPs in different doses have been observed for many representatives of this group of chemicals, at different dates of observation and routes of administration. In particular, this has been described for methyl parathion at a dose of 0.56 mg/kg (1/25 LD50)/1 time/3 days p/o for 8 weeks [17]; soman at a single dose of 0.67 LD50 [39]; dichlorophos at aerosol ingestion at a dose of 98.5 g/m^3^ with a daily exposure of 15 min for 28 days [40]; malathion at a dose of 75 mg/kg daily p/o for 7 days [41] and at a dose of 100 mg/kg daily p/o for 30 days [12]; diazinon once intraperitoneally in a dose of 100 mg/kg [10]; chlorpyrofos at a dose of 10 mg/kg combined with carbendazim at a dose of 50 mg/kg p/o for 7 days [42]; and some others. Thus, we can say that potential nephrotoxicity has been established for many OPs representatives if we take increased creatinine and urea concentrations as evidence of impaired renal function.

It is known that the concentration of calcium in blood plasma is about 2.5 mmol/L and about 60% of this amount (1.5 mmol/L) is in glomerular filtrate due to Ca^2+^ binding to proteins. Of the filtered amount of Ca^2+^ in the proximal tubules, up to 60% is reabsorbed; in the loop of Henle, about 30% is reabsorbed; and in the distal parts of the nephron, somewhere between 5 to 9% is reabsorbed. Ca^2+^ cations in the plasma are partially bound to proteins and are, thus, only partially filtered. Ca^2+^ is reabsorbed mainly intercellularly, passively through dense intercellular contacts or transcellularly (in distal tubules). The driving force of Ca^2+^ reabsorption in proximal tubules is a positive transcellular potential, i.e., the fluid of the tubule has a positive charge with respect to blood plasma. Disruption of this potential, due to POX poisoning and the induction of oxidative stress and associated impaired Ca^2+^ reabsorption, seems likely.

Inorganic phosphate (Pi) is found in plasma at pH 7.4 in the form HPO_4_^2−^ and H_2_PO_4_^−^ in the ratio 4:1. Both of these forms, similarly to glucose and amino acids, are mostly (two thirds) reabsorbed by secondary active transport in the proximal tubule via the NaPi-3 carrier, which is located on the apical membrane of epithelial cells and performs conjugated transport with Na^+^. Together with one phosphate molecule, three Na^+^ ions are transported into the cell. It is known that the reabsorption capacity of the kidney in relation to phosphate under normal conditions works on the “principle of overload”, due to which the excess of phosphate is rapidly excreted. In our studies, an increase in the content of phosphate in the blood plasma was observed only within 3 h after the poisoning, i.e., during the cholinergic crisis. Additional studies are needed to assess impaired NaPi-3 transporter function under the influence of POX and the contribution of the kidneys to the development of hyperphosphatemia.

Impaired alkaline phosphatase (ALP) activity, both in circulating blood and renal tissues, probably indicates that transphosphorylation reactions are impaired as a result of POX poisoning [43]. At the same time, changes in ALP content under the influence of OPs are quite variable depending on the dose and the route of entry into the body. Back in the 1980s, it was shown that administration of malathion to rats with different dietary protein levels resulted in different changes of ALP activity in kidney tissues: ALP increased at 5% and 20% dietary protein content, and decreased at 10% dietary protein [44]. Thus, the authors suggest that the interaction of OPs of pesticides with proteins is responsible for the degree of their toxicity, at least when they are ingested orally.

### 3.3. Urinalysis after POX Poisoning

Analysis of the urine of poisoned rats in the *M1/2* models did not reveal any significant signs of acute severe nephrotoxicity, such as proteinuria, decreased specific gravity, cylinduria, oliguria or polyuria, and hematuria. Urine pH and specific gravity in the poisoned animals also had no significant deviations from the control. However, a significant sign of impaired renal function in rats was a marked glucosuria observed 24 h after the poisoning in both the POX2x and CBPOX groups. Increased urinary excretion of glucose, protein, and blood is a marker of acute kidney damage [1]. As early as the 1960s, it was reported that glucosuria, proteinuria, hematuria, and oliguria were observed in rats intoxicated with parathion [45]. In a 2018 study, subchronic oral intoxication of rats with methyl parathion at a dose of 0.56 mg/kg for 8 weeks was also shown to result in a marked persistent hyperglycemia and glucosuria [17].

Glycosuria in rats observed in our studies 24 h after the poisoning is not accompanied by an increase in blood glucose levels, indicating its renal origin. It should be noted that glycosuria without concomitant hyperglycemia after OPs poisoning has also been described in humans [46,47]. The etiology of such glycosuria is not entirely clear, but renal tubule damage is considered to be one of the pathogenesis factors [46,48].

### 3.4. Biomarkers of Kidney Injury

In the *M1/2* models, when evaluating the content of markers of kidney damage in the urine of rats 3 days after the poisoning, an increase in calbindin excretion was found, though statistically significant changes were registered only in the CBPOX group (Table 1). Calbindin belongs to the group of calcium-binding proteins that were originally described as vitamin D-dependent calcium-binding proteins in the intestine and kidneys, where they are associated with TRPV5 channels and participate in reabsorption of Ca^2+^ ions [49,50]. In mammals, there are two main types, differing mainly in the size of the molecules with masses of 28 and 10 kDa and their distribution in tissues [51,52]. In the 1970s and 1980s, calbindin-D in human kidney was purified and identified as the 28 kDa form [53] and examined by IHC [54]. It has been demonstrated that calbindin-D predominantly localizes in the epithelial cells of the distal tubules of the kidneys and in the brain [55]. Calbindin levels in urine and serum have also been shown to increase in patients after extracorporeal shock wave lithotripsy [56,57] and after cisplatin therapy [58]. Currently, calbindin is considered as an informative biomarker of damage of distal tubules and collecting tubes of the kidneys [59,60]. In our studies in both *M1* (CBPOX) and *M3* (LD50) models of the poisoning, changes in the morphometric characteristics of the tubule epithelium were observed precisely in the early period after the poisoning, in 1–3 days, and together with increased calbindin excretion, indicating tubule damage or dysfunction.

Elevation of KIM-1 in the daily urine of POX2x and CBPOX rats 3 days after the poisoning is not statistically significant. KIM-1 is a transmembrane protein that is expressed at a low level in the normal kidney, but whose content is dramatically increased in kidney damage [61,62,63,64,65,66,67]. Thus, in both *M1* and *M2* models of the exposure, the levels of KIM-1 concentration in the urine of animals prevent us from concluding that the proximal tubules are severely damaged in rats after a single exposure to POX.

### 3.5. Creatinine Clearance in Rats after Exposure to POX

Creatinine is a near perfect substance for measuring glomerular filtration rate (GFR) [68]. Plasma creatinine is almost entirely a product of creatine and phosphocreatin metabolism in skeletal muscle [69,70]. With stable renal function, serum creatinine levels are usually constant, with variability in humans of about 8% per day [71]. Evaluation of endogenous creatinine clearance (ECC) in this regard can be used to estimate GFR, which was shown in experiments on rats as early as 1967 [72]. More recent studies have shown that, in five rat lines, including Wistar, creatinine clearance does not differ significantly from inulin clearance when measured or assessed simultaneously under comparable physiological conditions [73]. Currently, methods based on the use of endogenous creatinine content are being improved and new formulas convenient for calculating GFR have been proposed [74].

After the poisoning with POX, endogenous creatinine clearance in rats in the *M1/2* model decreased markedly within 24 h regardless of the mode of carboxylesterase inhibition. At the same time, 3 and 7 days after the poisoning, ECC in rats of all groups was not significantly different. This is evidence of a decrease in GFR in the early period after the poisoning, with subsequent recovery in the filtration level. A decrease in the effective RPF and GFR in rats after administration of POX at doses causing the development of systemic toxicity was demonstrated as early as in 1970 [26], and results of our studies agree with that publication. In humans, a decrease in ECC after OPs poisoning has also been registered, for example, with diazinon [75] and obidoxime [76].

### 3.6. Chondroitin Sulfate (CS) in Rat Urine after Exposure to POX

Glycosaminoglycans in the urine are 80% CS, 20% heparan sulfate, 1% to 2% dermatansulfate, and trace amounts of hyaluronic acid and keratan sulfate [77]. In the *M1/2* models, we found an increase in CS content in the daily urine of rats after 1 day. In particular, 3 days after the poisoning, those most pronounced in the CBPOX group were found. The results obtained concur with the results of our previous studies, in which we also found a significant increase in daily CS excretion with the urine of rats 1 and 4 days after poisoning by POX with a total dose of 275 µg/kg, which actually equaled LD84 [30]. Sulfated GAGs, which include CS, have a protective function. In the kidneys, they are the most important component of GBM, preventing the leakage of albumin and other proteins from the bloodstream due to electrostatic repulsion [78,79]. GAGs are synthesized by endothelial cells and podocytes [80,81], and they are localized in the *lamina rara* of basal membrane formed as a result of fusion of basal plates of podocytes and endothelial cells [78,79]. It should be noted that the role of CS both in OPs intoxication and in intoxication with other xenobiotics has been insufficiently studied. Based on our observations, the level of CS excretion with urine increases after poisoning at different doses of POX [27,30], as well as cyclophosphamide [82]. In this connection, at least for these two xenobiotics, a similar increase in CS excretion can be considered as a nonspecific marker of kidney (POX) and/or bladder (cyclophosphamide) damage.

### 3.7. Histopathological and Ultrastructural Changes in Rat Kidneys after Exposure to POX

Histopathological and ultrastructural changes in the kidneys, although they cannot explain the mechanisms of nephrotoxicity OPs, provide valuable information about the location and type of damage, which allows—in conjunction with other methods—assumption of the sequence of disorders and proposal of new components for adjuvant therapy.

The most common histopathological signs of OPs nephrotoxicity are damage and pathological changes in tubules. Thus, swelling and necrosis of epithelial cells, fatty degeneration of tubules, and capillary venous stasis of tubules were detected in rats that died after oral administration of parathion [50]. However, no significant changes were found in the kidneys of survived animals. There are published data on fatty degeneration of kidneys after soman and paraoxon administration [83]. Vacuolization of proximal tubule cells of monkey and rat kidneys has been described after carbaryl administration [84]. Parenchymatous dystrophy and vacuoles may explain the increased excretion of blood, glucose, and protein with urine [85]. Administration of parathion twice a week caused proliferation and fibrosis of the basal membrane of Bowman’s capsule and tubules; this was most pronounced in rats who received 8 mg/kg and survived for 200 days [1]. All these facts are most pronounced either when high doses of OPs are used or when it is administered chronically for a long time. Our studies demonstrate changes in rat kidney morphology even after single or double administration of POX.

In our study, we state the development of morphological changes in the rat kidneys within 3 days after the poisoning in the *M1/2* models and within 1 and 3 days in the *M3* model. These changes, including those characterized morphometrically, are observed in both models of poisoning, as can be seen when comparing the data of Table 2 and Table 3. Changes in tubule lumen diameter and epithelial cell height, as well as increased area of Bowman′s capsule and vascular glomeruli, are, in our opinion, obvious signs of OPs’ influence.

More pertinent for assessing the nephrotoxicity of OPs are the results of ultrastructural studies in the *M3* model. Although information on ultrastructural changes in the renal cells after OPs poisoning is very limited in the scientific literature [15,16], we can say that our results are in general agreement with it. We detected the partial destruction of cell apical surfaces, the filling of tubule lumen with cell destruction products, and the deformation of mitochondria 24 h after poisoning by POX. Damage to mitochondria is a key link mediating cell death. Mitochondria perform this function by releasing proapoptotic inducers and producing ATP, making it one of the main regulators of cell death by apoptosis, necrosis, or autophagy [86]. In terms of OPs nephrotoxicity mechanisms, mitochondrial damage is particularly important for the epithelial cells of the renal proximal tubules, whose cytoplasm contains a large number of mitochondria for active transport and secretion [87]. The establishment of deformation of mitochondria does not of course prove a violation of their functions after exposure to OPs. However, this could serve the starting point for the study of metabolic disorders in the mitochondria of renal epithelial cells using specific methods.

An interesting finding is the fact of basal membrane thickening in LD50 and LD84 groups 7 days after the poisoning. Absence of deposits on GBM and normal IHC reaction to nephrin in poisoned animals testify to the absence of early signs of podocyte damage. It is known that nephrin is a 180 kDa transmembrane protein expressed in glomerular podocytes and is a structural component of the slit diaphragm [88]. Nephrin molecules presented at the ends of podocytes in the form of a complex mesh, with a strong negative charge, repel the protein from penetrating into the Bowman space. The increased area of the renal corpuscles and the vascular glomerulus found in the *M1/2* models at 3 days after the poisoning, and 1 day after the poisoning at doses of LD50 and LD84 in the *M3* model, probably results from changes in blood pressure, namely RBF and GFR under the influence of POX. Other morphological changes in renal cell morphology after POX poisoning were not detected.

## 4. Materials and Methods

### 4.1. Animals

Male Wistar 240 rats (200–240 g) were kept in standard plastic T2 type cages, at +20–22 °C and light conditions of 12 h day–12 h night. Before the experiments, rats were acclimated to the forthcoming conditions for 96 h. The rats were fed with standard pelleted feed and water *ad libitum*. The non-pathogenic food for laboratory animals “Chara” was provided by Laboratorkorm company (Russia). The drinking water was purified according to recommendations of the Institute for Laboratory Animal Research (ILAR) and contained no more than 1 CFU/100 mL. Selection and randomization of animals were carried out by the comparative adaptive randomization method, and the tools of Graph Pad software (https://www.graphpad.com/quickcalcs (accessed on 15 November 2021). The study was conducted according to the EU Directive 2010/63/EU for animal experiments and approved by the Ethics Committee of the Sechenov Institute of Evolutionary Physiology and Biochemistry of the Russian Academy of Sciences (Ethical permit number 13-k-a, 15 February 2018).

### 4.2. Chemicals

All chemicals used in this work were of the highest purity available, from Sigma Aldrich (Saint Louis, MO, USA), Merck KGaA (Darmstadt, Germany), and other firms. Among those used were Paraoxon (D9286); Acid Fuchsin Orange G kit; (Bio-Optica^©^, Milano, Italy); TBS IHC Wash Buffer+Tween, 20× (Cell Marcue, Rocklin, CA, USA); detection system optimized for immuno-histochemistry (IHC) REVEAL-Biotin-Free Polyvalent DAB and REVEAL—Biotin-Free Pol-yvalent AP (Spring Bioscience, Pleasanton, CA, USA); MILLIPLEX MAP Rat Kidney Injury kit (Calbindin, KIM-1, TIMP-1); chondroitin 4-sulfate sodium salt from bovine trachea; anti-nephrin antidody produced in rabbit (PRS2265); osmium tetroxide 99.8% (201030); uranyl acetate (77870.02; Serva, Germany); and araldite 502. CBDP (2-(o-cresyl)-4H-1,3,2-benzodioxaphosphorin-2-oxide) was synthesized at the Research Institute of Hygiene, Occupational Pathology, and Human Ecology (RIHOPHE, St. Petersburg, Russia).

### 4.3. Three Models of Acute Intoxication of Rats by Exposure to Paraoxon

#### 4.3.1. Models 1 and 2 (*M1/2*). Acute Toxicity with Specific and Non-Specific Inhibition of Carboxylesterase Activity

When studying the mechanisms of action of OPs in toxicological experiments on rats, the principal problem is that a biochemical feature of blood plasma of this animal species is the presence of carboxylesterase activity. Mammalian carboxylesterases (CEs) belong to a multigenic superfamily with wide substrate specificity, and can catalyze the hydrolysis of esters, thioesters, amide-containing xenobiotics, and endogenous compounds (including fatty acid esters) [31,32]. In contrast to rodents and hares, there are no CEs in the blood plasma of humans, monkeys and mammalian horned ungulates [33]. Suppression of blood plasma CE activity in rodents, therefore, can significantly increase the adequacy of experimental models in studying the mechanism of action of OPs.

#### 4.3.2. Two Principles Were Used to Inhibit Carboxylesterase Activity (*M1* and *M2* Models)

In these models of exposure to POX, three groups of animals were formed: (1) intact animals (control)—12 rats; (2) POX2x group—22 rats (*M1 model*); and (3) CBPOX group—24 rats (*M2 model*). Preliminary, 60 min prior to administration of POX, administration of CBDP to the rats. Earlier, in a series of separate experiments, equitoxic doses of 110 µg/kg and 3.3 mg/kg of paraoxone and CBDP were selected, respectively. The main goal was to ensure that the applied CBDP dose did not inhibit the AChE of whole blood. It was found that whole blood AChE activity was more than halved 1 h after administration of POX at a dose of 110 µg/kg and CBDP at a dose of 4 mg/kg, whereas CBDP at a dose of 3.3 mg/kg and below did not inhibit AChE and BChE activity [27]. When CBDP (3.3 mg/kg) was administered, the second dose of POX to achieve LD50 was 165 µg/kg, i.e., CBDP administration increased rat sensitivity to POX. Double poisoning of rats with POX at doses of 110 and 130 µg/kg, as well as POX administration in a dose of 130 µg/kg one hour after CBDP administration at a dose of 3.3 mg/kg (CBPOX group), did not lead to animal death, but caused clinical manifestations of cholinergic crisis typical for OPs.

#### 4.3.3. Model 3 (*M3*). Acute Toxicity without Prior Inhibition of Carboxylesterase Activity

In contrast to models *M1/2*, the acute poisoning by POX in model *M3* caused clinical manifestations of cholinergic crisis and led to the death of a certain number of animals. In preliminary experiments, we determined the main toxicometric parameters of POX for male rats at a single p.c. injection: LD16 = 241 µg/kg, LD50 = 250 µg/kg, and LD84 = 259 µg/kg [27]. For experiments according to model 3 (*M3*), four groups of rats were formed through randomization: (1) intact animals (control)—10 rats; (2) LD16 group—8 rats; (3) LD50 group—14 rats; and (4) LD84 group—9 rats. To the rats of groups 2–4, POX was injected subcutaneously just once with the above mentioned doses.

### 4.4. Animal Observations and Sampling

Daily urine samples were collected in Techniplast^®^ Metabolic cages for Mice & Rats (Braintree Scientific, Braintree, MA, USA). At 1, 3, and 7 days after exposure to POX, a selective euthanasia of animals from each group and sampling of material for biochemical and histological studies were carried out. Animals were anesthetized by inhalation of isoflurane using a Univentor 410 Anesthesia Unit. A gas mixture containing isoflurane was fed into the animal chamber at a rate of 650–700 mL/min. The concentration of ioflurane in the chamber was 3.5–4%. After confirmation of signs of onset of anesthesia, the animals were decapitated using an Open Science rat guillotine.

After rat decapitation, heparinized blood samples (50 IU/mL) were centrifuged for 4 min at 1500× *g*. The resulting plasma was stored at −70 °C until analysis. The kidneys after euthanasia were quickly extracted and weighed, and tissue samples were excised for histological examination.

### 4.5. Biochemical Parameters of Blood Plasma

In the blood plasma of rats, 27 biochemical markers were determined: whole blood acetylcholinesterase (AchE), plasma cholinesterases (ChE), butyrylcholinesterase (BchE), plasma carboxylesterase (CE), paraoxonase 1 (PON1), alanine transaminase (ALT), albumin, total protein, glucose, D-3-hydroxybutyrate (3HB, beta-oxybutyric acid), triglycerides (TG), free fatty acids (FFA), glycerol, cholesterol, bile acids, high-density lipoproteins (HDL), low-density lipoproteins (LDL), lipase, iron, inorganic phosphate, alkaline phosphatase (ALP), urea, creatinine, calcium (Ca), uric acid (UA), amylase, and orosomucoid (alpha-1-acid glycoprotein, AAG). Blood plasma biochemical analyses were carried out after defrosting on an automated biochemical analyzer Sapphire 400 (Japan) using commercial kits according to manufacturer recommendations.

### 4.6. Urinalysis, Urine Biochemistry and Biomarkers of Renal Injury

Urinalysis was performed on the Combilyzer 13 analyzer (Human Diagnostics, Wiesbaden, Germany) using human-test Combina 13 strips. Urine specific gravity (USG) was additionally controlled using the Digital Hydrometer SBS-3500 (Menomonee Falls, WI, USA).

Sulphated glycosaminoglycans (GAGs) were determined in rat urine by the spectrophotometric method that we developed previously with 1.9-dimethyl-methylene blue [82].

Kidney injury biomarkers (Calbindin, KIM-1, TIMP-1) in urine were detected using a multiplex assay MagPix^®^ (Merck Millipore Corp., Danvers, MA, USA). To build calibration curves and determine analyte concentrations, xPONENT 4.2 software was applied.

### 4.7. Endogenous Creatinine Clearance

Creatinine was assayed in rat blood plasma and urine by an enzymatic method adapted for a 96-well plate on a Epoch microplate spectrophotometer (Biotek, Winooski, VT, USA) using a commercially available reagent kit (Randox, Crumlin, UK). Before determination, urine was diluted 1:49 by deionized water. Endogenous creatinine clearance (ECC) was calculated by the formula:

ECC = ([Ucr] × V)/([PCr] × T × m

where [Ucr]—urinary creatinine concentration, V—daily urine volume (output), [PCr] —average plasma creatinine concentration, T—urine sampling time, m—average rat body weight (g). ECC was expressed as mL/min/100 g rat body weight.

### 4.8. Histology, Immunohistochemistry (IHC) and Transmission Electron Microscopy (TEM)

Kidney tissue samples for a histological study were fixed in 10% buffered formalin solution for 24 h, dehydrated in ascending alcohol, and embedded in paraffin. The preparations were stained with hematoxylin and eosin.

Antibodies to nephrin (Sigma-Aldrich, Saint Louis, MO, USA) were used for immunohistochemical study. The antigen was demasked by heating in citrate buffer with pH 6.0 in a microwave oven, and the sections with antibodies were incubated in humid chamber at +4 °C for overnight. For antigen visualization we used the REVEAL-biotin-free polivalent AP detection system (Spring Bioscience Corp., Pleasanton, CA, USA).

The kidney tissue samples for the electron-microscopic study were fixed in 2.5% glutaraldehyde in phosphate buffer at pH 7.2–7.4 for 24 h, followed by fixation by 2% osmium oxide solution in phosphate buffer at pH 7.2–7.4 with sucrose. Successive dehydration was firstly used in ascending alcohols and then in acetone, prior to embedding in Araldit resin (Ted pella, Redding, CA, USA). Double contrasting ultrathin sections, 80 nm thick, were stained with lead nitrate solution and 1% aqueous uranyl acetate solution (Serva, Germany).

### 4.9. Visualization of Histological Preparations. Morphometry

The obtained preparations were examined at magnification modes 103×; 247× and 413×; 600×. Microscopes used include: Axiostar plus (Carl Zeiss, Oberkochen, Germany) with an image capturing device and monitor with lenses A-plan 10×/0.25, A plan 20×/0.45 ph 2, A plan 40×/0.65; light microscope Axio Imager A2 (Carl Zeiss, Oberkochen, Germany); and electronic microscope Merlin (Carl Zeiss, Oberkochen, Germany). Image analysis and morphometry were performed using Axio Vision 4.8.2 (Carl Zeiss, Oberkochen, Germany) and VideoTest Size 5.0 (Moscow, Russia).

### 4.10. Statistics

The data were processed using GraphPad Prism 5.0 software. Normality of distribution was checked using Kolmogorov–Smirnov and Shapiro–Wilk tests. Data with normal distribution were compared by one-factor ANOVA with Bonferoni correction. In other cases, the nonparametric Kruskal–Wallis test using Dunn criterion was applied for multiple comparisons. Two independent samples were compared by Mann–Whitney U-criterion. Differences were considered statistically significant at *p* < 0.05. Data are presented as mean ± standard deviation (M ± SD).

## 5. Conclusions

In studies using three experimental models of poisoning by POX, it has been shown that the nephrotoxic effects of POX in rats occur regardless of the mode of prior inhibition of carboxylesterase activity. The use of a conventional model of poisoning at a single dose of LD16 does not lead to the development of any abnormalities characterizing the nephrotoxicity of POX at this dose. At the same time, POX at the doses of LD50 and LD84 causes marked ultrastructural changes in the cells of tubules, which is a confirmation of its nephrotoxicity. Combined interpretation of the results with the data of biochemical study, biomarkers of renal damage and creatinine clearance suggests that, in the early period (1 to 3 days) after the poisoning, the changes registered are initially of afunctional nature, followed by development of changes in the structural elements of the nephron. Seven days after the poisoning, most of the revealed changes are normalized, but changes in GBM do not allow us to state that the consequences of poisoning are fully compensated.

## Figures and Tables

**Table 1:** Calbindin, KIM-1, and TIMP-1 in daily urine of rats 3 days after exposure to POX.

	Control	POX2x	CBPOX
Calbindin	4101 ± 334	6419 ± 1201	7383 ± 990 *
KIM-1	21.8 ± 5.5	28.8 ± 6.0	24.1 ± 6.9
TIMP-1	32,755 ± 7830	13,078 ± 9771	19,174 ± 11,596

* *p* ≤ 0.05; Results are presented in ng/mol_creatinine.

**Table 2:** Signs of POX nephrotoxicity in the *M1* and *M2* model.

	Time after Poisoning	Blood and Urine Biochemistry	Biomarkers of Kidney Injury	Histopathology
**POX2x**	24 h	**In plasma:** 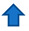 Uric acid **In urine:** 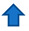 Glucose 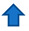 CS **Creatinine clearance:** 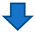 ECC	No change	No change
	3 days	**In urine:** 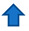 CS	No change	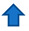 Renal glomerulus area 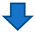 PCT lumen diameter
	7 days	**In plasma:** 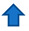 Uric acid	No change	No change
**CBPOX**	24 h	**In plasma:** 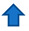 Creatinine 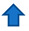 Calcium 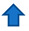 Inorganic phosphate 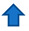 Alkaline phosphatase **In urine:** 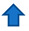 Glucose 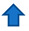 CS **Creatinine clearance:** 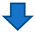 ECC	No change	No change
	3 days	**In urine:** 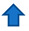 CS	**In urine:** 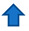 Calbindin	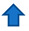 Renal glomerulus area 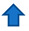 PCT lumen diameter 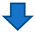 DCT lumen diameter
	7 days	No change	No change	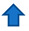 PCT lumen diameter

The “up” and “down” arrows indicate an increase or decrease in the index compared to the index of the control group rats.

**Table 3:** Signs of POX nephrotoxicity in the *M3* model.

	Time after Poisoning	Histopathology	Ultrastructural Changes
**LD16**	24 h	No	Not observed
	3 days	No	Not observed
	7 days	No	Not observed
**LD50**	24 h	Absence of granularity in the cytoplasm of tubule epitheliocytes; 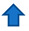 Expansion of Bowman’s capsule	Vacuolization of cytoplasm, degeneration of mitochondria of PCT epitheliocytes; 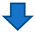 Decrease in epithelial cell height in DCT
	3 days	Absence of granularity in the cytoplasm of tubule epitheliocytes	Vacuolization of cytoplasm, degeneration of epithelial cell mitochondria in PCT
	7 days	Not observed	Blockage of tubule lumen by cellular detritus and local destruction of apical cell surfaces; 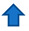 GBM thickening
**LD84**	24 h	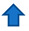 Dilation of the diameter of the lumen of the tubules; 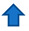 Expansion of Bowman’s capsule	Vacuolization of cytoplasm, degeneration of mitochondria of epitheliocytes in PCT karyopiknosis of nuclei; 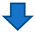 Decrease in epithelial cell height in DCT
	3 days	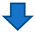 Narrowing of tubule lumen diameter	Vacuolization of cytoplasm, degeneration of mitochondria of epitheliocytes in PCT; 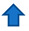 Increased height of the epithelium in the PCT
	7 days	Not observed	Partial blocking of tubule lumen by cellular detritus; local destruction of apical cell surfaces; 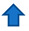 GBM thickening

The “up” and “down” arrows indicate an increase or decrease in the index compared to the index of the control group rats.

## Data Availability

The data presented in this study are available on request from the corresponding author.

## Abbreviations

AKI	Acute kidney injury
ALP	Alkaline phosphatase
AFOG	Acid Fuchsine Orange G
ARF	Acute renal failure
BW	Body weight
CBDP	2-(o-cresyl)-4H-1,3,2- benzodioxaphosphorin-2-oxide
CE	Carboxylesterase
CS	Chondroitin sulfate
DCT	Distal convoluted tubule
ECC	Endogenous creatinine clearance
GAG	Glycosaminoglycan
GBM	Glomerular basement membrane
GFB	Glomerular filtration barrier
GFR	Glomerular filtration rate
GSH	Glutathione
IHC	Immunohistochemistry
MODS	Multiple organ dysfunction syndrome
OPs	Organophosphates
PCT	Proximal convoluted tubule
Pi	Inorganic phosphate
POX	Paraoxon
PCr	Plasma creatinine concentration
RBF	Renal blood flow
TEM	Transmission electron microscopy
UCr	Urinary creatinine concentration
USG	Urine specific gravity
